# Supplementary material for: Evaluation of Novel Inhibitors of Tryptophan Dioxygenases for Enzyme and Species Selectivity Using Engineered Tumour Cell Lines Expressing Either Murine or Human IDO1 or TDO2
Source: Pharmaceuticals (Basel). 2022 Aug 31;15(9):1090. doi: 10.3390/ph15091090 (PMC9501369; doi:10.3390/ph15091090)
Supplement: Supplementary file 1 [file pharmaceuticals-15-01090-s001.zip › pharmaceuticals-1864710-supplementary.pdf]

Supplementary Table S1:

IDO1, IDO2 and TDO2 expression in 49 New Zealand melanoma lines before and 72 h after induction with human IFNγ.

Cell Line	IDO1		IDO2		TDO2	
	Non Induced	IFNγ	Non Induced	IFNγ	Non Induced	IFNγ
NZM 1	-	-	-	-	-	-
NZM 2	-	-	-	-	-	-
NZM 3	-	+	-	-	-	-
NZM 4	-	+	-	-	-	-
NZM 9	-	+	-	-	-	-
NZM 12	-	+	-	-	-	-
NZM 13	-	+	-	-	-	-
NZM 14	-	+	-	-	-	-
NZM 16	-	+	-	-	-	-
NZM 17	-	+	-	-	-	-
NZM 18	-	+	-	-	-	-
NZM 19	-	+	-	-	-	-
NZM 20	-	+	-	-	-	-
NZM 21	-	+	-	-	-	-
NZM 22	-	+	-	-	-	-
NZM 23	-	-	-	-	-	-
NZM 24	-	-	-	-	-	-
NZM 26	-	+	-	-	-	-
NZM 27	-	+	-	-	-	-
NZM 28	-	+	-	-	-	-
NZM 29	-	+	-	-	-	-
NZM 30	-	+	-	-	-	-
NZM 33	-	+	-	-	-	-
NZM 37	-	+	-	-	-	-
NZM 38	-	+	-	-	-	-
NZM 39	-	+	-	-	-	-
NZM 42	-	+	-	-	-	-
NZM 43	-	+	-	-	-	-
NZM 44	-	+	-	-	-	-
NZM 45	-	+	-	-	-	-
NZM 47	-	+	-	-	-	-
NZM 48	-	+	-	-	-	-
NZM 49	-	+	-	-	-	-
NZM 51	-	+	-	-	-	-
NZM 52	-	+	-	-	-	-
NZM 53	-	+	-	-	-	-
NZM 54	-	+	-	-	-	-
NZM 55	-	+	-	-	-	-
NZM 56	-	-	-	-	-	-
NZM 57	-	+	-	-	-	-
NZM 58	-	+	-	-	-	-
NZM 59	-	+	-	-	-	-
NZM 60	-	+	-	-	-	-
NZM 61	-	+	-	-	-	-
NZM 67	-	+	-	-	-	-
NZM 71	-	-	-	-	-	-
NZM 72	-	+	-	-	-	-
NZM 78	-	+	-	-	-	-
NZM 79	-	-	-	-	-	-
